# People with diabetes have a substantial lower life expectancty

**DOI:** 10.1093/eurpub/ckac131.128

**Published:** 2022-10-25

**Authors:** H Hilderink, R Poos, C Leemrijse, M Heins

**Affiliations:** 1 RIVM, Bilthoven, Netherlands; 2 NIVEL, Utrecht, Netherlands

## Abstract

**Background:**

People with diabetes live shorter lives on average than people without diabetes. This is not only because they have diabetes, but also more often other chronic diseases. This causes a greater risk of death. It makes a difference what kind of diabetes someone has. In type 1 diabetes, the natural immune system does not work properly. In type 2, an unhealthy lifestyle plays a role. An unhealthy lifestyle increases the chance that someone will get type 2 diabetes and the chance that that person will die of another disease, such as cardiovascular disease.

**Methods:**

RIVM has linked general practitioners’ data from 2012-2019 to the vital statistics of the Dutch National Statistical Office. The population of people registered with and without diabetes were coupled with the mortality data. Making use of life tables, associated life expectancies were calculated. For people under the age of 45, no robust analysis could be made.

**Results:**

People aged 45 with type 1 diabetes live on average 13 years shorter than people without diabetes. For a 45-year-old with type 2 diabetes, that is on average 4 years shorter. The chance of dying is about 5 times greater for people aged 45 to 60 with type 1 diabetes than for people without diabetes of this age. This difference becomes smaller as they get older, because people without diabetes also get one or more diseases more often. In type 2 diabetes, the mortality rate for people aged 45 to 60 years is about 2 times greater than for people without diabetes. Again, the difference decreases as they age.

**Conclusions:**

A substantial lower life expectancy of 13 years for type 1 and 4 years for type 2 diabetes has different societal and policy consequences. Since type diabetes 1 is unavoidable, more attention should be given to living with this condition, while for type 2 prevention of for example overweight should get attention.

**Key messages:**

• People with for type 1 and type 2 diabetes have 13 and 4 years lower life expectancy.

• More attention should be given to prevention and to living with diabetes.

